# WILLIAM ABRÃO SAAD: IN MEMORIAN TO THE GRAND MASTER OF HEPATIC SURGERY!

**DOI:** 10.1590/0102-672020210003e1588

**Published:** 2022-01-05

**Authors:** Marcelo A. F. RIBEIRO-JR, Paulo HERMAN, Luiz Augusto Carneiro D`ALBUQUERQUE, Fabrício F. COELHO, Eleazar CHAIB, Welington ANDRAUS, Ivan CECONELLO, William Abrão SAAD, Sérgio C. NAHAS

**Affiliations:** Professor Livre Docente da Disciplina de Cirurgia Geral e do Trauma da PUCSP-Sorocaba, Ex-Médico Colaborador do Serviço de Cirurgia de Fígado e Hipertensão Portal do HCFMUSP e Médico Colaborador do LIM 37 - Laboratório de Transplante e Cirurgia de Fígado da FMUSP, São Paulo, SP, Brasil; 2 Professor Associado do Departamento de Gastroenterologia da Universidade de São Paulo - FMUSP e Chefe do Serviço de Cirurgia de Fígado e Hipertensão Portal do HCFMUSP, São Paulo, SP, Brasil; 3 Professor Titular da Disciplina de Transplante de Fígado e Órgãos do Aparelho Digestivo do Departamento de Gastroenterologia da Universidade de São Paulo - FMUSP; São Paulo, SP, Brasil; 4 Assistente do Serviço de Cirurgia de Fígado e Hipertensão Portal do HCFMUSP, São Paulo, SP, Brasil; 5 Professor Associado do Departamento de Gastroenterologia da Universidade de São Paulo - FMUSP, São Paulo, SP, Brasil; 6 Assistente do Serviço de Transplante de Fígado e Órgãos do Aparelho Digestivo do HCFMUSP, São Paulo, SP, Brasil; 7 Professor Titular da Disciplina de Cirurgia do Aparelho Digestivo do Departamento de Gastroenterologia da Universidade de São Paulo - FMUSP, São Paulo, SP, Brasil; 8 Professor da Disciplina de Técnica Operatória da Universidade de Santo Amaro-UNISA, São Paulo, SP, Brasil; 9 Professor Titular da Disciplina de Coloproctologia do Departamento de Gastroenterologia da Universidade de São Paulo - FMUSP

William Abrão Saad was born on October 29, 1939, in the city of Araraquara, São Paulo, SP, Brazil. Their parents were Abrão Fadalala Saad and Carmen Habib Saad. He married Mrs. Ruth and had two children, William Abrão Saad Junior and Izabella Colane Barbosa Saad^1^.

He graduated from the Medical School of the University of São Paulo (FMUSP) Class of 1966. He specialized in surgery and devoted himself to a university career at the same educational institution where he graduated.

He had as main research lines digestive surgery and liver transplantation; portal hypertension and liver regeneration. He obtained his PhD title in Surgery, in 1972 and his Full professor of Surgery at the Department of Gastroenterology, in 1975, both at FMUSP.

He reached the position of Director of the Liver Surgery and Portal Hypertension Service at the Hospital das Clínicas of FMUSP, having pioneered the use of intraoperative ultrasound in this hospital, in addition to encouraging his team to implement the program for performing laparoscopic hepatectomies, a legacy that today puts the service under the leadership of Prof. Dr. Paulo Herman, as one of the great experiences of Latin America on the subject. He was a pioneer in the implementation of liver surgery in our midst in the most contemporary ways, and in addition he contributed a lot in the nineties with the strengthening of liver transplantation in our country.

Among other functions that he performed, the following stand out: Chairman at the Faculty of Medical Sciences of Sorocaba of the Pontifical Catholic University of São Paulo; professor of surgery at the School of Medicine of the University of Santo Amaro (Unisa); and professor of surgical technical skills at the Faculty of Medicine of the University of City of São Paulo (Unicid).

 William Abrão Saad served on the editorial board of the following journals: Arquivos Brasileiros de Cirurgia Digestiva (ABCD); Arquivos Brasileiros de Cirurgia Digestiva (ABCD); Revista do Colégio Brasileiro de Cirurgiões; Acta Cirúrgica Brasileira; Revista da Faculdade de Ciências Médicas de Sorocaba and Arquivos de Gastroenterologia.

He received dozens of awards during his career as a professor, being honored as a professor emeritus of Gastrão (Update Course in Surgery of the Digestive System, Coloproctology and Transplants) in 2012 and in 2014 he was honored for having implemented liver surgery in the Hospital das Clínicas complex at FMUSP and, as a man integrated in the activities for the benefit of the society, he was recognized as Cidadão Paulistano (Honorary Citizen from São Paulo) in 2009 project of authorship of the councilman Antonio Carlos Rodrigues.

William Abrão Saad was a member of the following entities: Brazilian College of Digestive Surgery and Asociaciones de Cirugia de Centro América y Panamá (honorary, 2004). He joined the São Paulo Academy of Medicine on April 2, 1982, rising to the status of an honorary member of this sodality. He was also honorary member of the Brazilian College of Surgeons.

He published 139 scientific articles in national and international journals; 55 abstracts in conference proceedings; more than 10 books, in addition to 60 chapters. He participated in 130 congresses and symposiums, having presented dozens of papers. He was a member of the organizing committee of six scientific events^2^^,^ 3.

He supervised nine master’s dissertations; three PhD theses and three other monographs. He produced 20 scientific videos. He participated in several examining boards, including 18 in public tenders; 27 master’s theses; two for doctoral qualifications; 17 doctorate; nine as a professor and eight as a full professor.

It is observed that its scientific legacy is outstanding and its contributions to national surgery are unquestionable. However, more than that, we allow ourselves to remember Professor Saad, the “Saadão” (big Saad), as a friend, teacher and for his talent to identify young talents in a unique way. Always easy to deal with, friendly, patient and tolerant, he respected everyone around him, treating them like family members. His good-natured way, always attuned to everything around him, made him a memorable figure. Who was not approached by him at the end of a lecture with the simple request: “... son, son, I have to give a lecture in Sorocaba on this topic, can I get a copy of your presentation??? Hence my friends, you know ...” It was a real chase until he had the material in hand: he uses to send his private driver at doctor office to wait, phone calls, etc., most of the times he didn’t even use it ... And if you owed a meeting certificate for him? It was better to owe money for sure!

His charisma and kindness allowed him to train dozens of disciples throughout Brazil. Many colleagues of national prominence in the hepatobiliopancreatic and transplant community were somehow supported by Professor Saad who was always ready to contribute to the Liver Surgery and Portal Hypertension Service at the FMUSP Hospital de Clínicas - where we can remember the professors Luiz Carneiro, Paulo Herman, Eleazar Chaib, doctors Vicenzo Pugliesi, Maria de Lourdes Capacci, Azzo Witman in São Paulo and in Sorocaba, professors Ronaldo Borghesi, José Mauro Rodrigues, Nelson Bocatto, Gisele Moreira, Angelo Zacariotto, Casio Rosas and Alvaro Gutierrez . In addition to these, other colleagues were at his side, such as me, Ben-Hur Ferraz Neto, Rogério Carballo Afonso, Osório Parra, Luigi Peduto, Adriano Pereira Sampaio, among many others.

During 32 years of personal contact, one of the authors of this editorial (MAFR) mentions, among hundreds of stories he has to tell, the fact that Professor Saad never refused to support and stimulate him mainly regarding his career at the university in the professional level. He recalls several occasions when Professor Saad volunteered to help him in emergency operations in the middle of the night without making any question - “stick to every job” -, resistant and always willing to work, which, by the way, was the that moved and stimulated him. More than 1500 patients operated together. Everything was done with him: thyroidectomies, gynecological, breast, gastroenterological, oncological procedures ..., that is, an old school general surgeon, of those that are not done nowadays. He mentions MAFR that he learned a lot, and here he thanks you, in memorian! He also mentions that he can witness his charisma, affection and respect with patients and family, his simple and welcoming way that allowed him to create a strong clinic during all his 55 years of professional practice. In the last surgical procedure in January 2021 shared with Professor Saad at São Camilo Hospital in São Paulo, MAFR and his friend Adriano Sampaio noticed signs of tiredness and back pain in him, but ... nothing that a Novalgina (dipyrone) and a glass of water could not solve ... He did not stop!

We will miss Professor Saad, his chats, his laughter at the operations and the lunches and dinners after them - which no one could ever try to pay -, from the messages on WhatsApp every day saying good morning and good night and sending “kisses …” , even of surprise cases, always more complex than they seemed and their simple way of facing challenges and obstacles.

Professor Saad is physically gone, but his memory and legacy will be with us forever!

See you soon Master!
